# Navigating Anesthesia for Thoracoscopic Biopsy in a Six-Year-Old Child With Eosinophilic Granulomatosis With Polyangiitis, Stroke Sequelae, and Pulmonary Collapse

**DOI:** 10.7759/cureus.93411

**Published:** 2025-09-28

**Authors:** Bheemas Atlapure, Satheesh Gunashekar, Sanjib Rawat, Bipul K Das, Habib Md R Karim

**Affiliations:** 1 Anesthesiology, Critical Care, and Pain Medicine, All India Institute of Medical Sciences, Guwahati, IND; 2 Cardiothoracic and Vascular Surgery, All India Institute of Medical Sciences, Guwahati, IND; 3 Pediatrics, All India Institute of Medical Sciences, Guwahati, IND

**Keywords:** blood hypereosinophilia, churg-strauss syndrome (css), collapse of one lung, granulomatosis with polyangiitis (gpa), pediatric-anesthesia, video-assisted thoracoscopic surgery (vats)

## Abstract

Eosinophilic granulomatosis with polyangiitis (EGPA), formerly known as Churg-Strauss syndrome, is a rare systemic vasculitis that affects small- to medium-sized vessels, characterized by asthma, hypereosinophilia, and multiorgan involvement. Pediatric cases are sporadic, and anesthetic management poses significant challenges due to airway hypersensitivity, allergy risk, and systemic complications. We describe the anesthetic management of a six-year-old girl with EGPA presenting with left-sided hemiparesis secondary to a prior intracerebral bleed, severe eosinophilia, and left lung collapse with pleural effusion. She was scheduled for video-assisted thoracoscopic surgery and pleural biopsy for persistent hypereosinophilia to confirm the probable diagnosis of EGPA. Considering her history of vasculitis, stroke, bronchial hyperreactivity, hypereosinophilia, and elevated IgE, the anesthetic plan prioritized avoidance of known allergenic agents such as neuromuscular blockers, opioids, non-steroidal anti-inflammatory drugs, and latex. General anesthesia was induced with propofol and sevoflurane. Although one-lung ventilation is often required, endotracheal intubation becomes a must, and it predisposes to pulmonary complications in such cases. A discussion with the surgical team was held to explore the use of both lung ventilation with low tidal volume and a supraglottic airway (i-gel®, Intersurgical Ltd, Berkshire, UK) to overcome the issues. Ultrasound-guided erector spinae block was administered as part of multimodal pain management. The procedure was limited to thoracoscopic biopsy as the need for further decortication was ruled out and was uneventful, and the patient had a stable recovery. This case underscores the importance of tailored anesthetic planning in pediatric EGPA patients with multisystem involvement. A multidisciplinary, individualized approach addressing hypersensitivity might ensure safe perioperative outcomes, even in complex cases involving the central nervous and respiratory systems.

## Introduction

Eosinophilic granulomatosis with polyangiitis (EGPA), formerly known as Churg-Strauss Syndrome, is a rare systemic vasculitis marked by asthma, eosinophilia, and vasculitis of small to medium-sized vessels. EGPA primarily affects adults, with a prevalence of 10 to 14 cases per million people and an annual incidence of 0.5 to 4.2 per million [1,2]. It typically presents with asthma, rhinosinusitis, peripheral eosinophilia, and systemic organ involvement, most frequently affecting the lungs, skin, and peripheral nervous system [3]. Central nervous system (CNS) involvement is rare but serious. Pediatric cases are exceedingly uncommon. Anesthetic management in these patients is particularly challenging due to their heightened allergic susceptibility, multisystem involvement, and airway hypersensitivity. This report describes the successful anesthetic management of thoracoscopic surgery in a six-year-old child with EGPA, CNS involvement, and unilateral lung collapse. The report highlights the importance of tailoring the anesthesia drugs and airway management. It demonstrates the feasibility of using a second-generation supraglottic airway device and dual lung ventilation during thoracoscopic surgery, where one-lung ventilation via endotracheal intubation is the standard method.

## Case presentation

A six-year-old girl (weight 20 kg, height 106 cm, body mass index 17.8 kg/m²) with a probable diagnosis of EGPA presented with a four-day history of headache and vomiting. She had a history of a hemorrhagic stroke one year prior, resulting in left hemiparesis. On physical examination, her vital signs were stable, with an oxygen saturation of 98% on room air and a respiratory rate of 22 breaths per minute. Chest auscultation revealed decreased air entry on the left side. Neurological examination revealed weakness (motor in the upper limb: proximal was 4/5 (right) and 3/5 (left); distal was 4/5 (right) and 2/5 (left), and in the lower limb: proximal was 4/5 (right) and 3/5 (left); distal was 4/5 (right) and 2/5 (left)). Deep tendon reflexes (knee and ankle) were brisk, with an extensor plantar response on the left side and spasticity in the left limbs, but no cranial nerve involvement was noted. She also reported mild respiratory discomfort.

Laboratory investigations revealed significant peripheral eosinophilia, with the most recent absolute eosinophil count (AEC) at 6.5 × 109/L, down from a previous peak of 48 × 109/L. The differential leucocyte count showed eosinophils at 47.9%, down from 58.3% in the last count (Table 1). Serum IgE levels were greater than 3000 IU/mL. Anti-nuclear antibody testing and sputum for acid-fast bacilli were negative.

**Table 1:** Hematological reports of the patient with their corresponding normal ranges. RBC: red blood cells, WBC: white blood cells.

Investigation	Result	Unit	Reference range
Hemoglobin	11.5	g/dl	11-14
Hematocrit	35.5	%	34-40
WBC count	21.15	x10^9^/L	5-15
Eosinophils	58.3	%	
Platelet count	317	x10^9^/L	200-490
RBC count	4.41	x10^12^/L	4.0-5.2
Absolute neutrophil count	2.71	x10^9^/L	1.5-8
Absolute lymphocyte count	5.73	x10^9^/L	6-9
Absolute monocyte count	0.33	x10^9^/L	0.2-1
Absolute eosinophil count	12.32	x10^9^/L	0.1-1
Serum IgE	>3000	IU/mL	0-440 (<7 years)

**Figure 1:**
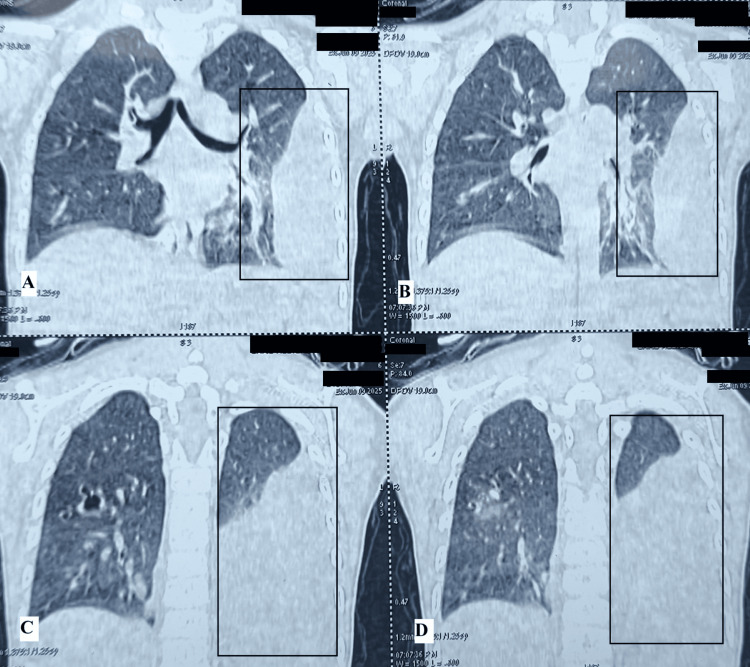
Sagittal view of baseline high-resolution computed tomography thorax shows left-sided pleural effusion, passive lung collapse, bronchiectasis, and ground-glass opacities in the highlighted box.

Magnetic resonance imaging of the brain showed gliotic changes from prior intraparenchymal hemorrhage and subacute subarachnoid hemorrhage. Other investigations ruled out infections, including tuberculosis. Chest computed tomography showed left-sided pleural effusion, passive lung collapse, bronchiectasis, and ground-glass opacities (Figure 1).

The child was suspected to have Churg-Strauss syndrome, meeting the American College of Rheumatology's criteria [4]. The other differential diagnoses were hypereosinophilic syndrome and parasitic infestation. Considering the patient is from a tuberculosis-endemic area and has a unilateral encysted pleural effusion, it was also considered in the differential diagnosis. She was started on ceftriaxone, albendazole, and ivermectin, but hypereosinophilia and symptoms persisted. Considering this and the need to begin precise therapy, the EGPA diagnosis needed to be confirmed, and tuberculosis needed to be excluded. Further, she was also in need of pleural drainage and, therefore, was scheduled for diagnostic video-assisted thoracoscopic surgery (VATS), pleural fluid drainage, and biopsy. Informed consent was obtained, with the parents being informed of the high-risk nature of the anesthetic plan.

Given the risk of hypersensitivity, non-latex gloves and equipment were used. A 22G IV cannula was in situ on the dorsum of the right wrist, and the child was premedicated with intravenous injections of glycopyrrolate (4 mcg/kg), midazolam (0.5 mg), and Ketamine (1 mg/kg) for a smooth transition to the operating room. Anesthesia was induced using 1.5 mg/kg propofol and inhaled sevoflurane (5% in 100% oxygen). A size-2 i-gel® (Intersurgical Ltd, Berkshire, UK) supraglottic airway was inserted without difficulty. Muscle relaxants were avoided entirely due to the risk of histamine release and potential cholinesterase deficiency. Anesthesia was maintained with sevoflurane (age-adjusted minimum alveolar concentration (MAC) 1.3-1.5) in a 50:50 air-oxygen mixture.

**Figure 2:**
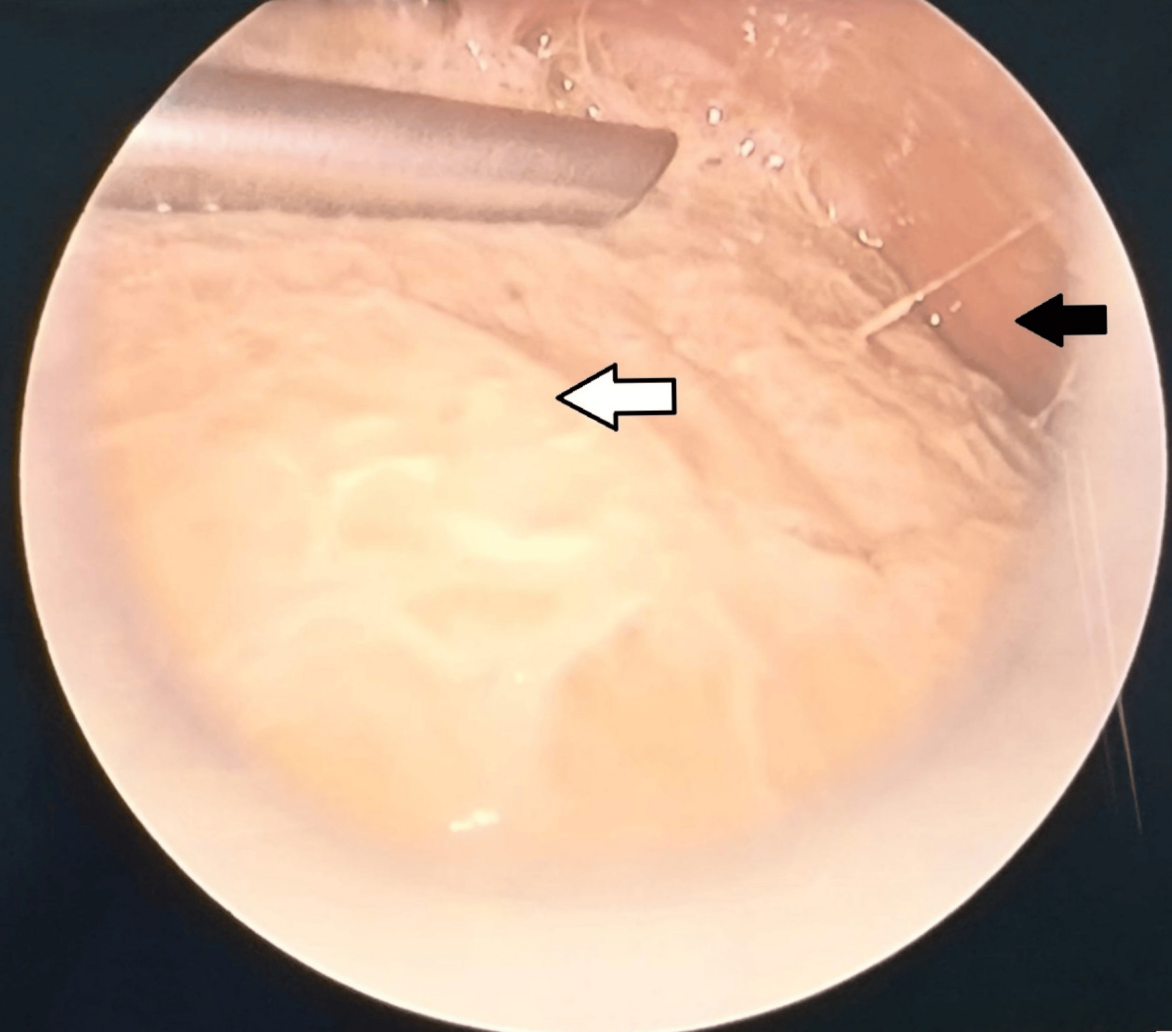
Intraoperative video-assisted thoracoscopic view, with a white arrow indicating thickened visceral pleura and a black arrow indicating seropurulent pleural fluid.

An ultrasound-guided erector spinae plane (ESP) block was administered at the D3 and D7 levels using a total of 15 mL 0.125% plain bupivacaine with 4 mg of dexamethasone in the right lateral decubitus position. Additional intraoperative medications included IV hydrocortisone (40 mg), tranexamic acid (200 mg), paracetamol (300 mg), pantoprazole (20 mg), and ondansetron (1.5 mg). Thoracoscopy revealed a thickened pleura and seropurulent collections (Figure 2); 50 mL of seropurulent fluid was aspirated. A 28F intercostal drain was placed following the procedure and closed.

**Figure 3:**
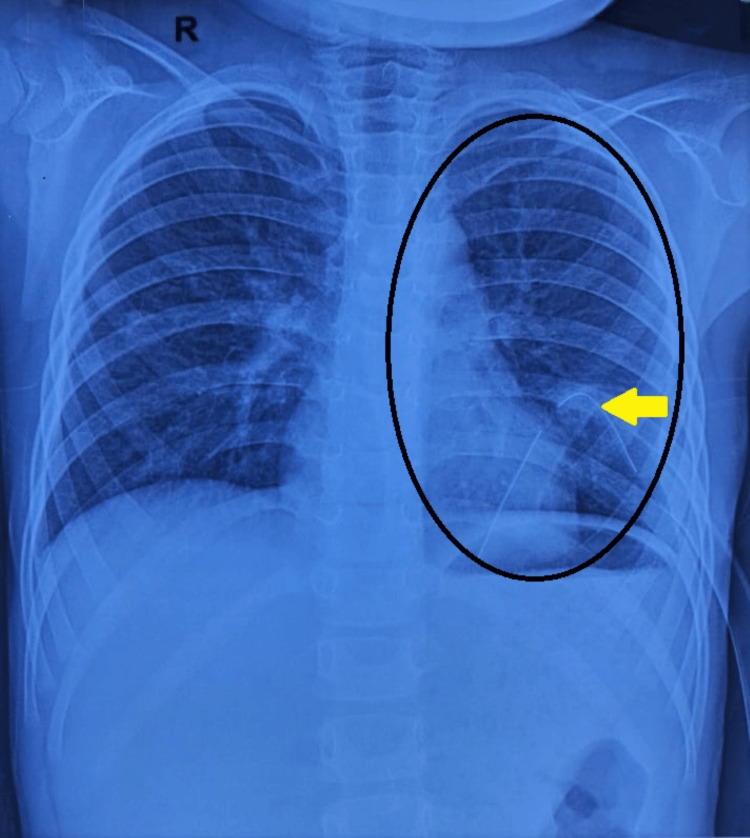
Post-operative posteroanterior view chest x-ray showing left lung expansion in the highlighted box, with the yellow arrow indicating the chest tube in situ.

The child was extubated in the right lateral position once spontaneous ventilation resumed with adequate tidal volumes. She remained hemodynamically stable throughout the procedure and was monitored in the post-anesthesia care unit for one hour before being transferred to the ward. Injection Streptokinase 5000 IU/Kg dissolved in 100 mL of 0.9% normal saline was instilled into the pleural cavity through the intercostal drainage tube and subsequently clamped for two hours, followed by drainage. This treatment was done on the third, fourth, and fifth post-operative days. A post-operative chest X-ray on day seven showed marked improvement in lung aeration (Figure 3).

The pleural fluid analysis showed a total leucocyte count of 19,115 cells/dL and a differential count of eosinophils 70%, neutrophils 10%, and lymphocytes 20%. The pleural biopsy from the left parietal pleura revealed features suggestive of eosinophilic pleuritis. 

## Discussion

This case illustrates the complexity of managing anesthesia in pediatric EGPA, particularly in cases with multisystem involvement and active pulmonary disease. EGPA diagnosis requires asthma, eosinophilia >1.5 × 10^6^/dl, and systemic vasculitis involving at least two organs [5]. Post-histopathologic examination of the pleura, our patient met all criteria. The updated 2022 American College of Rheumatology (ACR)/European Alliance of Associations for Rheumatology (EULAR) classification assigns a score of ≥6 with 85% sensitivity and 99% specificity for EGPA [6]. This patient had central nervous system (CNS) vasculitis with hemiparesis, marked eosinophilia, rhinosinusitis, pulmonary involvement, and elevated IgE (>3000 IU/ml).

Patients with EGPA are at high perioperative risk due to airway hyperresponsiveness and the potential for anaphylaxis. Severe perioperative allergic reactions are estimated to occur in one in 7000 to 10,000 cases, often due to latex, neuromuscular blocking agents (NMBAs), antibiotics, or chlorhexidine [7]. Although perioperative life-threatening anaphylaxis can even happen after a non-reactive skin test, antibiotics like vancomycin, azole group of anti-fungals, and some fluoroquinolones can even release histamine, cause reactions, and better be avoided [8]. In EGPA, latex and NMBAs are particularly concerning [9,10]. Latex-free surgical gloves and equipment were used. Muscle relaxants were avoided to prevent histamine release and potential cholinesterase deficiency [1,4,6,11]. Succinylcholine and cisatracurium, both known to trigger bronchospasm, were contraindicated [4].

Rocuronium, although commonly used, has been frequently implicated in anaphylaxis [10-12]. In our case, no muscle relaxants or reversal agents were used. Similarly, opioids and non-steroidal anti-inflammatory drugs were avoided due to their histaminergic and renal side effects. Instead, multimodal analgesia was employed, consisting of paracetamol, ESP block, and local infiltration.

Propofol and sevoflurane were chosen for their bronchodilatory properties and minimal histamine release [13]. These drugs are being successfully used for conducting GA in patients having hypereosinophilia in older children and adults [14]. Although our cases ended with thoracoscopic biopsy only, anesthesia was planned for VATS, including decortication. VATS often requires one-lung ventilation, thereby necessitating endotracheal intubation, which is also a stimulant for airway hypersensitivity. However, recent studies have shown that dual lung ventilation is also feasible for pathologies confined to the pleural cavity and pleura [15]. A supraglottic airway (i-gel®) was used to avoid the need for an endotracheal tube, which can cause tracheal stimulation. However, we had to keep the MAC slightly higher to prevent asynchrony, and muscle relaxants were also not used. Hydrocortisone was administered pre-induction to reduce mast cell degranulation and airway inflammation. The child was managed without complications related to the airway or allergies.

The French Vasculitis Study Group identifies five poor prognostic factors in EGPA: proteinuria >1 g/day, renal insufficiency, gastrointestinal involvement, cardiomyopathy, and CNS involvement [16]. Neurological involvement in EGPA patients can range from mild tingling and peripheral neuropathy to serious issues like confusion, seizures, central nerve palsy, and even life-threatening stroke; sensory symptom mainly presents first and even precede rash [17]. This child had a stroke and CNS sequelae, pulmonary collapse, asthma, and marked eosinophilia, indicating a poor prognosis. Nonetheless, safe perioperative and anesthetic outcomes were achieved through careful planning.

This report is limited by its single-patient design. Long-term neurological and pulmonary outcomes were not assessed. While anesthesia in EGPA is described in adults and even in children, reports on children undergoing VATS or thoracoscopic pleural biopsy that also use a supraglottic airway are either lacking or scarce. Therefore, even though it is a single case, anesthetic drugs are well-tested in other situations; the present report contributes scientifically credible insights into perioperative anesthetic considerations for pediatric EGPA patients undergoing thoracic procedures.

## Conclusions

Pediatric EGPA with CNS and pulmonary involvement poses significant anesthetic challenges. Avoidance of known allergens, drugs associated with histamine release, use of supraglottic airway devices with dual lung ventilation, steroid prophylaxis, and regional anesthesia techniques as key components of multimodal analgesia, form a crucial part of a successful anesthetic strategy. This case underscores the importance of multidisciplinary planning in high-risk pediatric cases and offers valuable insights into anesthetic management in rare vasculitic disorders.
